# Insights into Novel Viral Threats in Sweetpotato from Burkina Faso: Characterisation of Unexplored Pathogens

**DOI:** 10.3390/v17091222

**Published:** 2025-09-07

**Authors:** Pakyendou E. Name, Ezechiel B. Tibiri, Fidèle Tiendrébéogo, Seydou Sawadogo, Florencia Djigma, Lassina Traoré, Angela O. Eni, Justin S. Pita

**Affiliations:** 1 Laboratoire de Virologie et de Biotechnologies Végétales, Institut de l’Environnement et de Recherches Agricoles (INERA), Ouagadougou 01 BP 476, Burkina Faso; 2 Laboratoire de Biologie Moléculaire et de Génétique (LABIOGENE), Université Joseph KI-ZERBO, Ouagadougou 03 BP 7021, Burkina Faso; 3 Central and West African Virus Epidemiology (WAVE), Pôle Scientifique et d’Innovation de Bingerville, Université Félix Houphouët-Boigny (UFHB), Bingerville 01 BP V34, Côte d’Ivoire

**Keywords:** alternative hosts, nanopore sequencing, sweetpotato, circular DNA viruses, satellites, Burkina Faso

## Abstract

Sweetpotato is a key staple crop in tropical and subtropical regions. Its vegetative propagation makes it a persistent reservoir, facilitating the emergence and spread of complex infections. Understanding its virome is crucial for disease management and food security. We investigated the sweetpotato virome in Burkina Faso using rolling circle amplification and Oxford Nanopore sequencing. Eight symptomatic leaf samples, previously undiagnosed using conventional methods, were analysed. Bioinformatic pipelines were employed followed by phylogenetic comparisons. Two viruses known to infect sweetpotato, namely sweet potato leaf curl virus (SPLCV) and sweet potato leaf curl deltasatellite 3 (SPLCD3), were consistently detected in all samples. Additionally, pepper yellow vein Mali virus (PepYVMV), cotton leaf curl Gezira alphasatellite (CLCuGeA) and cotton leaf curl Gezira betasatellite (CLCuGeB) were identified for the first time in this crop. Phylogenetic analysis confirmed their genetic proximity to isolates from tomato, okra and pepper. Their co-occurrence with SPLCV and SPLCD3 indicates a complex viral landscape that could influence disease severity. This study highlights the underestimated role of sweetpotato as a viral reservoir, influencing virus evolution and transmission. Further studies should assess their pathogenicity, co-infection dynamics and vector-mediated transmission to improve crop productivity.

## 1. Introduction

Sweetpotato (*Ipomoea batatas* [L.] Lam) is a perennial crop grown worldwide, particularly in tropical and subtropical regions. It is rich in carbohydrates, fiber, vitamins and minerals, especially beta-carotene [1,2,3]. Due to its high yielding potential and ability to grow in a wide range of environments, sweetpotato has established its place as a strategic crop for food security in more vulnerable regions [4,5,6]. It also contributes to subsistence farming and trading, providing income for smallholders while diversifying agricultural production.

However, sweetpotato present a major inconvenience as they are propagated vegetatively, which promotes the vertical accumulation and propagation of viruses within populations and contributes to the persistence of complex virome [7]. More than 30 viruses infecting sweetpotato have been identified worldwide [5,8,9]. These viruses often occur as complexes in mixed infections, leading to disease syndromes such as sweet potato virus disease (SPVD), which can cause severe yield losses estimated at up to 90% in some cases [10,11]. The perennial nature of sweetpotato, combined with vegetative propagation, favors virus adaptation, interaction, and dissemination [9,12,13], while also increasing the likelihood that emerging or previously undetected viruses are maintained and spread.

In Burkina Faso, several sweetpotato viruses have been reported, including SPFMV, SPCSV, and SPLCV, primarily via NCM-ELISA, PCR-based assays targeting known viruses, cloning and Sanger sequencing [9,14]. However, some symptomatic samples could not be fully characterised, although grafting onto *Ipomoea setosa* consistently reproduced infection-like symptoms. Attempts to obtain full-length SPLCV clones via rolling circle amplification (RCA) following cloning were unsuccessful, as enzymatic digestion of the RCA products revealed distinct patterns. This observation strongly suggests the presence of additional viral agents that remain undetected by conventional methods. The increasing recognition of single-stranded circular DNA viruses as emerging and re-emerging plant pathogens further supports the possibility that these agents may also be involved in sweetpotato infections in this region. These observations underscore the need for more comprehensive diagnostic strategies.

High-throughput sequencing (HTS) has emerged as a powerful method for deciphering the complex viral communities associated with sweetpotato in Burkina Faso. Over the last decade, several HTS-based studies have contributed to the identification of viruses not previously recorded on certain crops or species [15]. For instance, the detection of pepper yellow vein Mali virus (PepYVMV) on tobacco in Burkina Faso [16] and melon chlorotic spot virus (MeCSV) in cultivated sorrel in Belgium [17] have revealed unsuspected natural alternative hosts. Similarly, tomato chlorosis virus (ToCV) has been identified in tomatoes and other economically important crops, including cowpea, eggplant, lettuce, potato, squash and sweet pepper [18].

Building on these advantages, the present study investigated the sweetpotato virome in Burkina Faso using HTS, with a particular focus on single-stranded circular DNA viruses. We report the characterisation of three previously unsuspected or unrecorded virus and satellites associated with sweetpotato in Burkina Faso. These findings expand our understanding of the viral complexes affecting this strategic food security crop and provide new insights into the evolutionary dynamics and spread of single-stranded circular DNA viruses within African agroecosystems and beyond.

## 2. Materials and Methods

### 2.1. Screening of Study Samples

**Figure 1:**
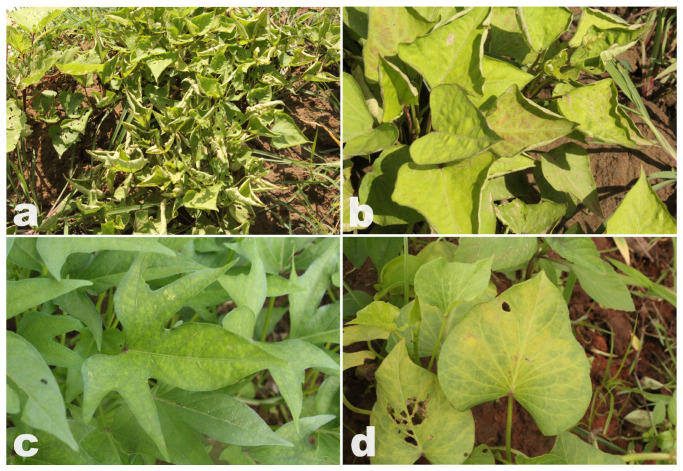
Leaf samples of sweetpotato exhibiting some symptoms of sweet potato virus infection. (**a**,**b**) leaf curling and deformation (moderate and severe, respectively); (**c**) mosaic; (**d**) yellowing.

**Figure 2:**
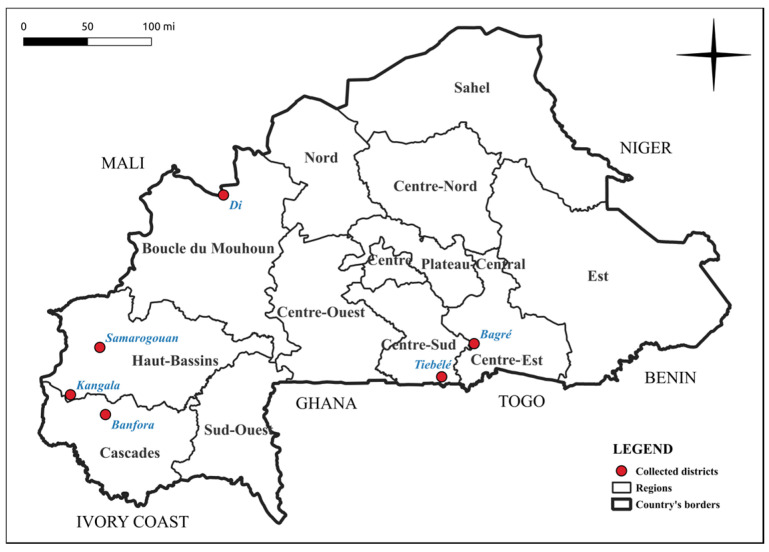
Map of Burkina Faso highlighting districts where selected samples were collected.

Samples selected in this study were screened from an extensive bank of biological samples collected in Burkina Faso over two consecutive years, 2015 and 2016, as part of a previous study [9]. The leaf samples were dried and well preserved at INERA’s ‘‘Laboratoire de Virologie et de Biotechnologie Végétale (LVBV)’’ in Kamboinsé, Ouagadougou, Burkina Faso. These samples, which showed typical symptoms such as yellowing, mosaic, leaf curling and leaf deformation (Figure 1) were selected for further investigation due to limited detection achieved by conventional molecular assays and cloning experiments, as described in the Introduction section. For this study, eight of these samples were selected due to the potential presence of uncharacterised pathogens. Specially, samples BFA310 and BFA313 originated from Tiébélé, BFA351 and BFA362 from Bagré, BFA942 from Samorogouan, BFA1094 from Banfora, BFA407 from Di, and BFA662 from Kangala (Figure 2).

### 2.2. Extraction of Nucleic Acids and Enrichment

Dried leaves of four samples were ground using TissueLyser II (QIAGEN, 40724 Hilden, Germany); and total DNA were extracted with cetyltrimethylammonium bromide (CTAB) following the protocol described by Doyle and Doyle [19]. The quantity and quality of nucleic acids were assessed using a Nanodrop 2000c spectrophotometer (Thermo Scientific, Wilmington, DE, USA). Then approximately 100 ng of total DNA were amplified by the rolling circle amplification (RCA) using the TempliPhi DNA polymerase DNA amplification kit (Amersham Biosciences Corp., Sunnyvale, CA, USA) as described in Inoue-Nagata et al. [20].

### 2.3. Library Preparation and Nanopore Sequencing

Native Barcoding 96 V14 (SQK-NBD114.96, Oxford Nanopore Technologies, Oxford, UK) was used to prepare libraries according to the manufacturer’s instructions. The main steps involved in preparing the DNA libraries included digestion with T7 Endonuclease I (New England Biolabs, MA, USA, Cat. No. M0302L), end-prep, barcode ligation, and adapter ligation. Initially, RCA products were digested using T7 Endonuclease I (New England Biolabs, MA, USA, Cat. No. M0302L) by combining the purified RCA amplicon (approximately 1 µg) with T7 Endonuclease I (New England Biolabs, MA, USA, Cat. No. M0302L), and Invitrogen™ DEPC-treated Water (Thermo Fisher Scientific, MA, USA, Cat. No. AM9906), followed by incubation at 37 °C for 30 min. Digested DNA is then purified using AMPure XP Beads (Beckman Coulter, CA, USA, Cat. No. A63881) and quantified with an Invitrogen™ Qubit™ 4 Fluorometer (Thermo Fisher Scientific, Illkirch, France), using Invitrogen™ Qubit™ dsDNA HS Assay Kit (Thermo Fisher Scientific, Illkirch, France, Cat. No. Q32854). Next, 200 ng of DNA was end-repaired and dA-tailed using the NEBNext Ultra II End repair/dA-tailing module (New England Biolabs, MA, USA, Cat. No. E7546L). After incubation at 20 °C for 5 min and 65 °C for 5 min, DNA was purified and the native barcodes were bind. Barcoded samples are pooled and purified, then 200 ng is processed for adapter ligation using NEBNext Quick Ligation Module (New England Biolabs, MA, USA, Cat. No. E6056S) and Quick T4 DNA Ligase (New England Biolabs, MA, USA, Cat. No. M2200S). The library was purified, quantified, and prepared (50 fmol) for sequencing by loading onto a SpotON Flowcell and sequenced on a MinION device (Mk1C, Oxford Nanopore Technologies, Oxford, UK).

### 2.4. Bioinformatics Analysis

The raw data (POD5 format) were basecalled using the DORADO tool (v0.7.2) in super-accurate basecalling mode (“SUP”) (https://github.com/nanoporetech/dorado, accessed on 18 June 2024). The resulting FASTQ reads were demultiplexed, and adapter sequences were removed using Porechop (v0.2.4) (https://github.com/rrwick/Porechop, accessed on 21 June 2024). Quality control and summary statistics were assessed with NanoPlot (v1.42.0) [21]. Reads were cleaned by mapping them against the host reference genome (*Ipomoea batatas*, GenBank accession GCA_002525835) and the human genome (GRCh38.p14) using Minimap2 (v2.17) [22]. Taxonomic assignment was performed using Kraken2 (v2.0.8) [23] to quickly and accurately classify sequences to the appropriate taxonomic level, allowing for a comprehensive assessment of viral diversity. The cleaned reads were then assembled de novo using Flye (v2.9.3) [24], an assembler optimised for long ONT reads. The resulting contigs were then polished using Medaka (v1.12.0) (https://github.com/nanoporetech/medaka, accessed on 08 July 2024) to refine the assembly by correcting residual errors, thereby improving the overall quality of the reconstructed genome. These sequences were subsequently annotated using Prokka (v1.14.6) (https://github.com/tseemann/prokka, accessed on 20 March 2025) and submitted to the GenBank. Sequences were analysed using the BLAST+2.16.0 search tool (https://blast.ncbi.nlm.nih.gov, accessed on 20 March 2025) against the NCBI’s database to identify closely related viruses.

### 2.5. PCR Screening and Sanger Sequencing

A PCR was performed using the degenerate primer pair VD360 (5′-AGRCTGAACTTCGACAGC-3′) and CD1266 (5′-TCTCAACTTCARGGTCTG-3′), as described by Delatte et al. [25] to confirm the presence of pepper yellow vein Mali virus (PepYVMV) detected by nanopore sequencing. PCR was performed in a 20 µL reaction containing 4 µL of FIREPol Blend Master Mix (Solis BioDyne, Tartu, Estonia), 0.5 µL of each primer (10 µM), 1 µL of DNA (100 ng), and 14 µL of nuclease-free water. Amplification conditions were as follows: an initial denaturation at 94 °C for 4 min, followed by 35 cycles of denaturation at 94 °C for 45 s, annealing at 55 °C for 55 s, elongation at 72 °C for 60 s, and a final elongation step at 72 °C for 7 min. PCR products were analyzed by 1% agarose gel electrophoresis in 0.5X TAE buffer, stained with ethidium bromide, and visualized using a BIORAD Gel Doc XR+ System (Marshall Scientific, NH, USA). The amplified PCR products were then sent to Macrogen^®^ (Amsterdam, Netherlands) for sequencing.

### 2.6. Phylogenetic Analysis

Homologous sequences were retrieved from GenBank (Table 1) to establish phylogenetic relationships between our isolates and strains available in the NCBI database. All sequences were aligned using the ClustalW tool integrated into MEGA 11 [26], using default parameters to ensure optimal global alignment. The best-fitting model for phylogenetic tree construction was determined in MEGA 11 using the Models/Method option. For the PepYVMV isolates, the Tamura-Nei model with gamma distribution was used. For the cotton leaf curl Gezira alphasatellite (CLCuGeA) and cotton leaf curl Gezira betasatellite (CLCuGeB) isolates, the Tamura 3-parameter model with gamma distribution was used. The phylogenetic tree was constructed using the maximum likelihood (ML) method in MEGA 11, based on the selected substitution models. Support values were evaluated using 1,000 bootstrap replicates to ensure robust branch reliability. The resulting phylogenetic tree was visualized and annotated using FigTree v1.4.4 (https://github.com/rambaut/figtree, accessed on 24 March 2025) to facilitate interpretation of phylogenetic relationships. The pairwise nucleotide (nt) and amino acid (aa) sequence identities between the isolates in this study and their homologous sequences were also calculated.

## 3. Results

### 3.1. Viral Genome Assembly

A total of 1,976,102 reads were generated across the eight samples, resulting in a data volume of 2.04 Gb. The mean read length ranged from 920.3 bp to 1148.2 bp, with N50 values ranging from 1361 to 2031 bp. Mean quality scores (Q-scores) ranged from 13.4 to 14.6, which is in line with performance expectations for the Oxford Nanopore Technologies platform. The majority of reads were relatively short length, with a marked concentration near the origin. A sharp decrease in the number of reads was observed as read length increased. Average quality scores were predominantly between 10 and 20, with only a small proportion of reads exceeding a Q-score of 25. These results underscore the robustness and reliability of the raw sequencing data, ensuring its suitability for subsequent analysis and downstream applications.

After de novo assembly, BlastN and BlastX comparisons of the contigs obtained with the GenBank database showed that all eight samples were infected with at least three or four viruses. Two viruses known to infect sweetpotato, namely sweet potato leaf curl virus (SPLCV) and sweet potato leaf curl deltasatellite 3 (SPLCD3), were consistently detected in all samples, confirming their widespread presence in the production regions of Burkina Faso. In addition to these ubiquitous viruses, other begomovirus and satellites were identified. PepYVMV was found in Tiébélé samples (BFA310 and BFA313). CLCuGeA was detected in samples from Bagré (BFA351), Di (BFA407) and Kangala (BFA662), while CLCuGeB was mainly found in samples from Bagré (BFA362), Samorogouan (BFA942) and Banfora (BFA1094).

This distribution highlights clear profiles of co-infection across different geograhical districts, underlining the complex virome structure associated with sweetpotato in Burkina Faso (Table 2). Due to the unexpected presence of PepYVMV, CLCuGeA and CLCuGeB in sweetpotato, subsequent analyses focused specifically on these viruses, aiming to characterise their genomes and phylogenetic relationships.

### 3.2. Sequence Analysis of Assembled Viral Genomes

For isolate [BF:Tie:BFA310:16], a partial contig of 2128 bp was obtained, showing 100% query coverage and 86.80% nucleotide identity with PepYVMV isolate MH778656 that was previously reported in tomato (*Solanum lycopersicum*). Similarly, isolate [BF:Tie:BFA313:16] produced a partial 2551 bp contig that shared 85.09% identity and full coverage with the same reference sequence. These results suggest that the circulating virus strain in this region is relatively conserved. Additionally, the complete genome of PepYVMV (2781 bp) was reconstructed, with 99.17% identity and full coverage to isolate MH778658 (also isolated on the tomato), supported by high sequencing depth (5578.1×), which further confirms its prevalence and conservation.

The presence of PepYVMV was confirmed by PCR amplification using the VD360/CD1266 primer pair, followed by Sanger sequencing. The coat protein (CP) sequences obtained showed 100% nucleotide identity with those initially detected by Nanopore sequencing, confirming the accuracy and consistency of the initial detection (see Supplementary Table S1).

For the CLCuGeA, several isolates showed close relationships with known sequences. For example, isolate [BF:Bag:BFA351:16] yielded a complete contig of 1353 bp with 96.61% nucleotide identity and 100% query coverage to the Burkina Faso isolate MK032296, originally isolated from okra (*Abelmoschus esculentus*). Similarly, isolate [BF:Di:BFA407:16] produced a contig of identical length with 96.90% identity and 100% coverage to the same reference. Another isolate, [BF:Kan:BFA662:16], produced a contig of 1353 bp with 97.71% nucleotide identity and 100% query coverage to the okra yellow crinkle Cameroon alphasatellite isolate (OkYCrCMA) isolate Njo5sp2 (FN675287), indicating a wider regional distribution of this satellite DNA.

For the CLCuGeB, isolates showed significant similarity to known sequences from Burkina Faso. Isolate [BF:Bag:BFA362:16] yielded a complete contig of 1348 bp with 97.78% identity and 99.77% query coverage with isolate FN554574, previously identified in okra. Similarly, isolate [BF:Sam:BFA942:16] produced a 1333 bp contig with 96.52% nucleotide identity and 100% coverage to isolate FN554576. Finally, isolate [BF:Ban:BFA1094:16] reconstructed a 1,348 bp contig also with 97.78% identity and 100% coverage to the same isolate FN554576. The assembly depth of 188.5× for this isolate further supports the accuracy of the analysis.

De novo assembly and complete genome statistics for the isolates obtained are shown in Table 3.

### 3.3. Genomic Organization and Phylogenetic Relationships of PepYVMV

**Figure 3:**
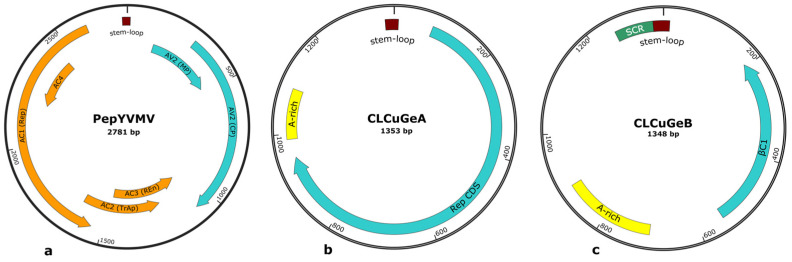
Genomic organization and detailed annotation of isolates characterised in this study. (**a**) genome of PepYVMV includes the following annotated regions: C1 (Rep), encoding a replication-associated protein; C2 (TrAp), encoding a transcription-activating protein; C3 (REn), encoding a replication-activating protein; V1 (CP), encoding a capsid protein; and V2 (MP), encoding a movement protein. (**b**) genome of CLCuGeA contains Rep CDS, encoding a replication protein, and an A-rich region, which is an adenine-rich sequence. (**c**) genome of CLCuGeB includes βC1, which encodes a betasatellite-specific protein; the SCR (Satellite Conserved Region); and an A-rich region, an adenine-rich sequence.

The complete genome of the PepYVMV-BFA942 isolate (PV405580), characterised in sample BFA942 (Samorogouan) and confirmed by Sanger sequencing, has a length of 2781 bp and displays the typical organization of begomoviruses (Figure 3). Annotation reveals a total of six open reading frames (ORFs): two ORFs in the viral sense (AV1 and AV2) and four ORFs in the complementary sense (AC1, AC2, AC3 and AC4). In the viral direction, AV1 is 777 nt and encodes a capsid protein (CP), containing 258 amino acids (aa). AV2, 351 nt, encodes a movement protein (MP) of 116 aa. AV1 and AV2 overlap by around 200 nt. In the complementary direction, AC1, 1,065 nt in size, encodes a 354-aa replication-associated protein (Rep). AC2 (408 nt) encodes a 135-aa transcription-activating protein (TrAp). AC3, with a size of 405 nt, encodes a replication-activating protein (Ren) of 134 aa. AC2 overlaps with AC1 and AC3; and AC4 which is, included in AC1, is 258 nt in size and codes for an 85 aa protein. The genome also features a 307-nucleotide non-coding intergenic region (IR), located between the AC1 and AV2. This region contains a specific genomic structure, a stem-loop structure located near the origin of replication. This structure includes the conserved non-nucleotide sequence 5′-TAATATT↓AC-3′.

**Figure 4:**
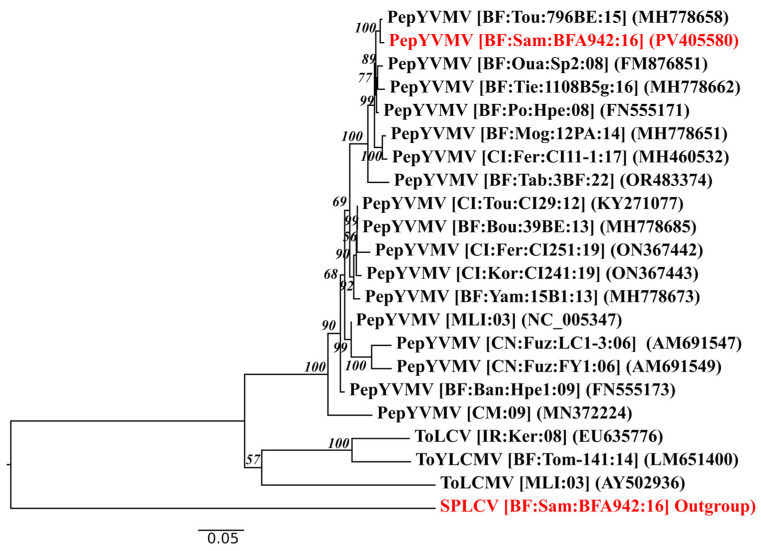
Maximum likelihood phylogenetic tree of PepYVMV isolate characterised in this study. Isolates from this study are in red, and GenBank references are in black. The phylogenetic tree is rooted with an isolate of SPLCV characterised in this same sample as PepYVMV. Abbreviations: SPLCV, sweet potato leaf curl virus; PepYVMV, pepper yellow vein Mali virus; ToLCV, tomato leaf curl virus; ToLCMV, tomato leaf curl Mali virus; ToYLCV, tomato yellow leaf curl Mali virus.

Phylogenetic analysis of the PepYVMV isolate (Figure 4) revealed that the sequences from Burkina Faso, including PepYVMV-BFA942 (PV405580), PepYVMV-796BE (MH778658), PepYVMV-Oua:Sp2 (FM876851), and PepYVMV-Mog:12PA:14 (MH778651), clustered within a strongly supported monophyletic clade. This indicates a high degree of genetic homogeneity among these isolates and suggests a recent common ancestry.

Sequences from neighbouring countries, such as Côte d’Ivoire (CI:Fer:C11-1:17, MH460532; CI:Kor:CI241:19, ON367443) and Mali (MLI:03, NC_005347), formed distinct but adjacent clades, reflecting moderate divergence while maintaining regional relatedness. This may have been shaped by local ecological and agricultural factors.

In contrast, sequences from China (AM691547, AM691549) formed a distinct group, demonstrating significant phylogenetic divergence from the African isolates. More distantly related begomoviruses, including ToLCV and SPLCV, clustered in separate clades.

These phylogenetic patterns were corroborated by nucleotide and amino acid sequence analysis, which revealed high similarity (≥97%) among Burkina isolates, confirming their strong genetic homogeneity (Supplementary Table S2). Identities of over 95% with West African reference sequences further support close evolutionary relationships within the region. In contrast, more distant sequences from Asia and Europe exhibited moderate to low identities (78–94%), and the outgroup displayed markedly low similarity (~57–59%), consistent with their placement in distant clades.

### 3.4. Genomic Organization and Phylogenetic Relationships of CLCuGeA

Complete genomes of CLCuGeA isolates (PV405574-PV405576), characterised from samples BFA351 (Bagré), BFA407 (Di) and BFA662 (Kangala), have a length of 1353 bp and display the typical organization of alphasatellites. Annotation (Figure 3) revealed a secondary structure, the stem-loop, located near the origin of replication and containing the conserved nonanucleotide motif 5′-CAGTATT↓AC-3′. The genome comprises a single ORF, Rep, in the viral direction, 870 nt in size, encoding a replication protein of 289 amino acids. An adenine-rich region (A-rich), located between the stem-loop and the Rep ORF, comprises 51% of A residues, a characteristic of satellite viruses.

**Figure 5:**
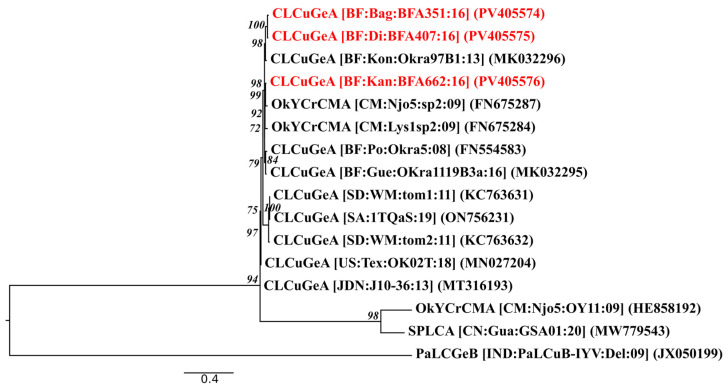
Maximum likelihood phylogenetic tree of CLCuGeA isolates characterised in this study. Isolates from this study are in red, and GenBank references are in black. The phylogenetic tree is rooted with an isolate of papaya leaf curl Gezira betasatellite (PaLCGeB; GenBank accession JX050199). Abbreviations: CLCuGeA, cotton leaf curl Gezira alphasatellite; OYCrCMA, okra yellow crinkle Cameroon alphasatellite.

Pairwise nucleotide and amino acid identity analysis (see Supplementary Table S3) revealed that most CLCuGeA isolates shared a high similarity (>92%), with the Burkina isolates clustering at over 97%. This indicates a strong genetic relationship within this group. This was reflected in the maximum likelihood phylogenetic tree (Figure 5), in which the Burkina isolates formed a distinct monophyletic clade. These isolates were positioned close to isolates from Cameroon (OYCrCMA), which formed an adjacent clade. However, the relationships between these two groups are supported by lower bootstrap values, suggesting moderate phylogenetic divergence, that may reflect geographic or ecological differences.

In contrast, isolates from Sudan and Saudia Arabia formed well-supported clades, emphasising the existence of distinct regional lineages that are likely to have been shaped by local adaptation. Further away in the tree, sequences such as HE858192 (Cameroon) and MW779543 (China) (~50% identity) and JX050199 (India) (~36%) branched separately from the West African group, which is consistent with their low pairwise identities. Overall, the results demonstrate strong genetic cohesion among West African isolates while clearly distinguishing them from CLCuGeA populations circulating in other parts of Africa and Asia.

### 3.5. Genomic Organization and Phylogenetic Relationships of CLCuGeB

Annotation of complete genomes of CLCuGeB (PV405577-PV405579), characterised in sample BFA1094 (Banfora), BFA362 (Bagré) and BFA942 (Samorogouan) revealed typical features of betasatellites (Figure 3). The genome features in the complementary sense, a single ORF, with a size of 354 nt. It codes for the βC1 protein composed of 117 amino acids. The genome also includes a stem-loop structure with the begomovirus non-nucleotide sequence 5′-TAATATT↓AC-3′. Upstream of the stem-loop structure is a satellite conserved region (SCR), characteristic of betasatellites. In addition, an adenine-rich region (*A-rich*), located between positions 703 and 891 nt, contains around 58% A residues, similar to what is observed in alphasatellites.

**Figure 6:**
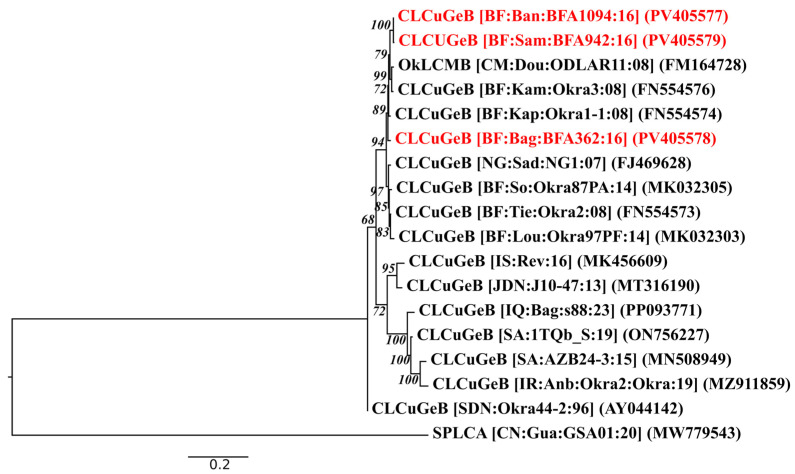
Maximum likelihood phylogenetic tree of CLCuGeB isolates characterised in this study. Isolates from this study are in red, and GenBank references are in black. The phylogenetic tree is rooted with an isolate of sweet potato leaf curl alphasatellite (SPLCA; GenBank accession FM164729). Abbreviations: CLCuGeB, cotton leaf curl Gezira betasatellite; OkLCMA, okra leaf curl Mali alphasatellite.

Pairwise nucleotide and amino acid identity analysis (see Supplementary Table S4) revealed a high degree of similarity (>97%) among the Burkina CLCuGeB isolates (PV405577-PV405579), indicating strong genetic homogeneity within this group. These isolates also exhibited high sequence similarity (>96%) with other Burkina isolates, such as CLCuGeB-Kam:Okra3:08 (FN554576) and CLCuGeB-Kap:Okra1:08 (FN554574), as weel as the Cameroon isolate OkLCMB (FM164728). This high level of identity is reflected in the phylogenetic tree (Figure 6), in which the three isolates from this study formed a strongly supported monophyletic clade closely related to other sequences from West Africa. This clustering suggests a recent common ancestor and limited regional variability, which is likely to be maintained under relative stable agroecological conditions and agricultural practices.

Isolate from Niger, such as NG:Sad:NG1:07 (FJ469628), was positioned in adjacent subclade and displayed high sequence similarity with the Burkina group. This supports the hypothesis of genetic exchange between these neighbouring regions, possibly via overlapping vector populations or shared cropping systems.

In contrast, isolates from geographically distinct regions, including Israel (MK456609), Saudi Arabia (MN508949), and Sudan (AY044142), formed distinct clades, which is consistent with their lower pairwise identities (83-93%). The Sudanese isolate (AY044142) remained closer to the West African cluster (~93% identity), suggesting partial relatedness despite its phylogenetic separation. Further divergence was observed in isolates from Iran (MZ911859) and other Asian regions, which formed distinct clades and exhibited moderate sequence similarity (83–89%) with West African isolates.

## 4. Discussion

The study identified three viruses and two satellites, including SPLCV and SPLCD3 (previously reported in sweetpotato) and three unexpected in this crop: PepYVMV, CLCuGeA and CLCuGeB. The consistent detection of SPLCV and SPLCD3 in all samples confirms that these viruses are well-established in sweetpotato in Burkina Faso [9]. Their presence aligns with previous studies and highlights their widespread distribution across major production areas. While our study did not focus on these viruses, their detection supports earlier findings and provides context for the overall viral community in sweetpotato. In contrast, the detection of PepYVMV, CLCuGeA and CLCuGeB represents unexpected viral associations, indicating the need for further investigation into their emergence in sweetpotato and their epidemiological significance in the region. While these viruses have been documented in other host plants, such as okra, tomato, pepper and tobacco [16,27,28,29,30,31,32], their identification in sweetpotato provides evidence that their host range may include this crop, expanding our understanding of their ecological adaptability. Importantly, these detections were not made in earlier studies that used conventional PCR-based diagnostics on the same samples collected in Burkina Faso [9]. These diagnostics rely on prior knowledge of target sequences and assume that only known viruses infect the crop in question. In the case of sweetpotato, diagnostic efforts have traditionally focused on a limited number of well-characterised viruses that have already been reported in this crop. This approach excludes other viral agents that have not previously been suspected or studied in this context. Compared to previous studies, the use of Oxford Nanopore sequencing in our study enabled more complete and accurate viral characterisation, highlighting the presence of PepYVMV, CLCuGeA and CLCuGeB in sweetpotato. These results demonstrate the usefulness of high-throughput sequencing technologies for characterising viral communities and improving the detection of known and previously unreported viruses in sweetpotato.

Unlike conventional diagnostic methods that are limited to detecting known pathogens, HTS provides a comprehensive and flexible approach. By analyzing the entirety of the metagenomic data present in a sample, HTS allowed us to obtain an initial overview of the sweetpotato virome. This has been demonstrated both in our study and in other studies [33,34,35], highlighting its particular relevance in agricultural systems where pathogen interactions can influence symptoms and increase yield losses. Free from biases introduced by prior knowledge, this technology is a powerful tool for advancing our understanding of viral diversity and enhancing disease management strategies for crops. However, HTS does not directly reveal recombination events or the ability of viruses to coexist with other pathogens. To better characterise these features, genomic-based comparative analyses, such as phylogenetic methods and recombination detection tools, are required.

Phylogenetic analyses confirmed the relevance of this approach, revealing high genetic similarity between the CLCuGeA and CLCuGeB isolates identified in this study and those previously reported in okra and tomato in Burkina Faso [27,31]. These results may indicate localized spread or a common infection source, consistent with the high genetic similarity observed between isolates and potentially facilitated by shared vectors. Similarly, the PepYVMV-BFA942 isolate closely resembles isolates identified on pepper and tobacco in the same country in previous studies [16,29,32]. The phylogenetic patterns may reflect regional viral circulation, as related isolates were observed in neighbouring countries such as Niger, Côte d’Ivoire, and Mali, suggesting possible transboundary dynamics. Furthermore, our findings align with earlier observations that viral species show no genetic structuring based on the host plant, reflecting their ecological adaptability and potential for cross-host transmission [27,31]. The high sequence similarity of PepYVMV-BFA942 isolate with previously reported isolates from okra, tomato, pepper and tobacco suggests that the detection of these viruses in sweetpotato is not the result of recent introductions. Instead, they probably reflect well-established viral lineages that are already circulating in Burkina Faso and the wider West African region.

The identified alphasatellites and betasatellites are typically associated with cotton leaf curl Gezira virus (CLCuGeV) [27,30,31,36]. To our knowledge, this study represents the first report of CLCuGeA and CLCuGeB on sweetpotato in Burkina Faso and globally. Similarly, PepYVMV, previously described in hosts such as okra, tomato, and tobacco, is now first reported in sweetpotato through the isolate PepYVMV-BFA942 (PV405580). These findings highlight the need for further studies to improve our understanding of these viruses’ host range and their mechanisms of adaptation to sweetpotato. Such studies would also help us to assess their potential impact on tuber quality and agronomic performance. They also open up new avenues for investigating the role of these viruses in viral complexes involving well-known pathogens such as SPLCV.

Alphasatellites and betasatellites, though dependent on begomoviruses for encapsidation and transmission by *Bemisia tabaci*, exhibit distinct biological roles [37]. Betasatellites, for example, are key drivers of symptom induction in infected plants through the βC1 protein, a RNA silencing suppressor that helps viruses evade host immune defenses [38,39]. In contrast, alphasatellites are generally considered symptom attenuators, although some have shown similar gene-silencing suppression mechanisms [40,41]. Together, these satellites can significantly influence the impact of viral infections on host crops.

Begomoviruses, including PepYVMV, play a central role in the dynamics of viral complexes in agriculture. They are known for their ability to infect a wide range of hosts and induce diverse symptoms such as chlorosis, leaf curling, and severe yield losses [42,43,44]. Notably, PepYVMV has shown high adaptability to different hosts, such as okra, tobacco, and now sweetpotato, due to its close interaction with vectors like *Bemisia tabaci* [16]. The plasticity of this virus, combined with its interactions with satellites and other pathogens like SPLCV, highlights its critical role in shaping viral complexes that can influence symptom severity and potentially impact agricultural productivity [45].

Agricultural practices in Burkina Faso contribute significantly to the spread of these viruses. Crop rotation, as well as intercropping with several crops in association on the same plot, which is common in some agro-ecological zones, often includes crops such as sweet potato, okra, tomato and cereals, which can serve as alternative hosts for these pathogens. While crop rotation is beneficial for managing pests (e.g., aphids, and whiteflies) [46] and improving yields [47], it can also create conditions that favor the persistence and spread of viral complexes. The high mobility of vectors like *Bemisia tabaci* is a key factor in spreading begomovirus-associated viruses and satellites [48,49,50,51]. These vectors, attracted to different crops, facilitate the cross-transmission of viruses, thereby increasing the diversity of infected plants and the persistence of pathogens in agroecosystems.

The porosity of borders in Africa amplifies the dynamics of viral spread [52]. Weak phytosanitary controls at border entry points and the unregulated exchange of plant materials (cuttings, seeds, and tubers) create ideal conditions for long-distance viral dissemination [53,54]. This is particularly problematic for vegetatively propagated crops like sweetpotato, where latent pathogens can be introduced into new regions. Informal exchanges of planting materials among farmers, without proper health inspections, further exacerbate this issue, enabling the durable establishment of viruses in previously unaffected areas.

The findings suggest that sweetpotato acts as a significant reservoir or accumulator of viruses. This capacity, combined with local agricultural practices and vector mobility, could explain the persistence and complexity of viral infections observed. While these viruses have been genetically characterised, further studies are needed to assess their pathogenicity on sweetpotato, their impact on productivity, and their interactions with other pathogens, particularly SPLCV. Future research should also explore integrated management strategies, including epidemiological monitoring, the development of resistant varieties, and vector control measures.

The present study provides substantial evidence that sweetpotato functions as a substantial reservoir for a wide array of viral pathogens, encompassing both well-documented viruses and recently identified circular DNA viruses. The perennial nature of sweetpotato, its vegetative propagation practices, and the pervasive informal exchange of planting material across regions contribute to its remarkable capacity to accumulate and maintain viral populations [9]. To further elucidate the biological significance of these associations, we strongly recommend targeted pathogenicity assays specifically grafting and vector-mediated inoculation to evaluate each virus’s capacity to induce disease symptoms in sweetpotato and other susceptible crops. In parallel, controlled co-infection experiments will be critical for determining whether synergistic effects exacerbate symptom severity, compromise tuber yield and quality, or interact with established pathogens such as SPLCV. Conducting field trials under diverse agroecological conditions will provide robust data on yield losses attributable to these viral complexes, thereby clarifying their economic impact. Finally, comprehensive genomic analyses that investigate recombination events and genetic diversity within these viral populations will illuminate their evolutionary potential and guide the development of effective, long-term disease management strategies.

## 5. Conclusions

In this study, we identified three novel viruses infecting sweetpotato in Burkina Faso: PepYVMV, CLCuGeA, and CLCuGeB. These findings represent, to the best of our knowledge, the first documented occurrences of these pathogens on sweetpotato worldwide. Our findings underscore the potential role of sweetpotato as a viral reservoir, facilitating the persistence and dissemination of diverse viral complexes within agroecosystems. Further investigation is needed to confirm these findings and to evaluate the effects of these viruses on sweetpotato yield, tuber quality, and susceptibility to other pathogens. In particular, elucidating the mechanisms of viral adaptation to sweetpotato, assessing co-infections with SPLCV, and clarifying the epidemiological impact of vectors such as *Bemisia tabaci* will be critical. By integrating controlled pathogenicity assays, field trials, and advanced genomic analyses, future research can provide a more complete understanding of how these new viral threats affect sweetpotato production and long-term crop health.

More broadly, this work highlights the importance of proactive surveillance and robust diagnostics for emerging viruses in underexplored regions like Sub-Saharan Africa. By deepening our knowledge of viral diversity and transmission dynamics, we can develop more effective disease management strategies for sweetpotato and support food security initiatives in vulnerable agricultural systems.

## Figures and Tables

**Table 1:** Isolates from this study (bold) and GenBank references used for phylogenetic tree construction.

Virus Isolate	Strain	Host	Geographical Origin	Accession No.
**[BF:Sam:BFA942:16]**	**Pepper yellow vein Mali virus**	***Ipomoea batatas***	**Burkina Faso**	**PV405580**
[BF:Tou:796BE:15]	Pepper yellow vein Mali virus	*Solanum lycopersicum*	Burkina Faso	MH778658
[BF:Po:Hpe:08]	Pepper yellow vein Mali virus	*Capsicum frutescens*	Burkina Faso	FN555171
[BF:Tie:1108B5g:16]	Pepper yellow vein Mali virus	*Sida acuta*	Burkina Faso	MH778662
[BF:Oua:Sp2:08]	Pepper yellow vein Mali virus	*Capsicum annuum*	Burkina Faso	FM876851
[BF:Mog:12PA:14]	Pepper yellow vein Mali virus	*Capsicum frutescens*	Burkina Faso	MH778651
[CI:Fer:CI11-1:17]	Pepper yellow vein Mali virus	*Solanum melongena*	Ivory Coast	MH460532
[BF:Tab:3BF:22]	Pepper yellow vein Mali virus	*Tobacco*	Burkina Faso	OR483374
[CI:Tou:CI29:12]	Pepper yellow vein Mali virus	*Capsicum sp*	Ivory Coast	KY271077
[BF:Bou:39BE:13]	Pepper yellow vein Mali virus	*Solanum lycopersicum*	Burkina Faso	MH778685
[CI:Kor:CI241:19]	Pepper yellow vein Mali virus	*Solanum lycopersicum*	Ivory Coast	ON367443
[CI:Fer:CI251:19]	Pepper yellow vein Mali virus	*Capsicum frutescens*	Ivory Coast	ON367442
[BF:Yam:15B1:13]	Pepper yellow vein Mali virus	*Capsicum annuum*	Burkina Faso	MH778673
[BF:Ban:Hpe1:09]	Pepper yellow vein Mali virus	*Capsicum frutescens*	Burkina Faso	FN555173
[MLI:03]	Pepper yellow vein Mali virus	*-*	Mali	NC_005347
[CN:Fuz:LC1-3:06]	Pepper yellow vein Mali virus	*Eclipta prostrata*	China	AM691547
[CN:Fuz:FY1:06]	Pepper yellow vein Mali virus	*Bougainvillea*	China	AM691549
[CM:09]	Pepper yellow vein Mali virus	*Malvastrum*	Cameroon	MN372224
[IR:Ker:08]	Tomato yellow leaf curl virus	*Lycopersicon esculentum*	Iran	EU635776
[BF:Tom-141:14]	Tomato yellow leaf curl Mali virus	*Solanum lycopersicum*	Burkina Faso	LM651400
[MLI:03]	Tomato leaf curl Mali virus	*-*	Mali	AY502936
**[BF:Sam:BFA942:16]**	**Sweet potato leaf curl virus**	***Ipomoea batatas***	**Burkina Faso**	**This study**
**[BF:Bag:BFA351:16]**	**Cotton leaf curl Gezira Alphasatellite**	***Ipomoea batatas***	**Burkina Faso**	**PV405574**
**[BF:Di:BFA407:16]**	**Cotton leaf curl Gezira Alphasatellite**	***Ipomoea batatas***	**Burkina Faso**	**PV405575**
**[BF:Kan:BFA662:16]**	**Cotton leaf curl Gezira Alphasatellite**	***Ipomoea batatas***	**Burkina Faso**	**PV405576**
[SD:WM:tom1:11]	Cotton leaf curl Gezira alphasatellite	*Solanum lycopersicum*	Sudan	KC763631
[SA:1TQaS:19]	Cotton leaf curl Gezira alphasatellite	*Solanum lycopersicum*	Saudi Arabia	ON756231
[SD:WM:tom2:11]	Cotton leaf curl Gezira alphasatellite	*Solanum lycopersicum*	Sudan	KC763632
[JDN:J10-36:13]	Cotton leaf curl Gezira alphasatellite	*Okara*	Jordan	MT316193
[US:Tex:OK02T:18]	Cotton leaf curl Gezira alphasatellite	*Abelmoschus esculentus*	USA	MN027204
[BF:Kon:Okra97B1:13]	Cotton leaf curl Gezira alphasatellite	*Abelmoschus esculentus*	Burkina Faso	MK032296
[BF:Gue:OKra1119B3a:16]	Cotton leaf curl Gezira alphasatellite	*Abelmoschus esculentus*	Burkina Faso	MK032295
[BF:Po:Okra5:08]	Cotton leaf curl Gezira alphasatellite	*Abelmoschus esculentus*	Burkina Faso	FN554583
[CM:Njo5:sp2:09]	Okra yellow crinkle Cameroon alphasatellite	*Abelmoschus esculentus*	Cameroon	FN554587
[CM:Lys1sp2:09]	Okra yellow crinkle Cameroon alphasatellite	*Abelmoschus esculentus*	Cameroon	FN675284
[CM:Njo5:OY11:09]	Okra yellow crinkle Cameroon alphasatellite	*Abelmoschus esculentus*	Cameroon	HE858192
[CN:Gua:GSA01:20]	Sweet potato leaf curl alphasatellite	*Ipomoea batatas*	China	MW779543
[IND:PaLCuB-IYV:Del:09]	Papaya leaf curl Gezira betasatellite	*Ipomoea purpurea*	India	JX050199
**[BF:Bag:BFA362:16]**	**Cotton leaf curl Gezira betasatellite**	***Ipomoea batatas***	**Burkina Faso**	**PV405578**
**[BF:Sam:BFA942:16]**	**Cotton leaf curl Gezira betasatellite**	***Ipomoea batatas***	**Burkina Faso**	**PV405579**
**[BF:Ban:BFA1094:16]**	**Cotton leaf curl Gezira betasatellite**	***Ipomoea batatas***	**Burkina Faso**	**PV405577**
[CM:Dou:ODLAR11:08]	Okra leaf curl Mali virus betasatellite	*Abelmoschus esculentus*	Cameroon	FM164728
[BF:Kam:Okra3:08]	Cotton leaf curl Gezira betasatellite	*Abelmoschus esculentus*	Burkina Faso	FN554576
[BF:Kap:Okra1-1:08]	Cotton leaf curl Gezira betasatellite	*Abelmoschus esculentus*	Burkina Faso	FN554574
[BF:So:Okra87PA:14]	Cotton leaf curl Gezira betasatellite	*Abelmoschus esculentus*	Burkina Faso	MK032305
[BF:Tie:Okra2:08]	Cotton leaf curl Gezira betasatellite	*Abelmoschus esculentus*	Burkina Faso	FN554573
[NG:Sad:NG1:07]	Cotton leaf curl Gezira betasatellite	*Abelmoschus esculentus*	Niger	FJ469628
[BF:Lou:Okra97PF:14]	Cotton leaf curl Gezira betasatellite	*Abelmoschus esculentus*	Burkina Faso	MK032303
[SDN:Okra44-2:96]	Cotton leaf curl Gezira betasatellite	*Abelmoschus esculentus*	Sudan	AY044142
[IS:Rev:16]	Cotton leaf curl Gezira betasatellite	*Solanum lycopersicum*	Israel	MK456609
[JDN:J10-47:13]	Cotton leaf curl Gezira betasatellite	*Okara*	Jordan	MT316190
[IQ:Bag:s88:23]	Cotton leaf curl Gezira betasatellite	*Abelmoschus esculentus*	Iraq	PP093771
[SA:1TQb_S:19]	Cotton leaf curl Gezira betasatellite	*Solanum lycopersicum*	Saudi Arabia	ON756227
[IR:Anb:Okra2:Okra:19]	Cotton leaf curl Gezira betasatellite	*Abelmoschus esculentus*	Iran	MZ911859
[SA:AZB24-3:15]	Cotton leaf curl Gezira betasatellite	*Solanum lycopersicum*	Saudi Arabia	MN508949
[CN:Gua:GSA01:20]	Sweet potato leaf curl alphasatellite	*Ipomoea batatas*	China	MW779543

**Table 2:** Viral co-infection profiles in sweetpotato samples analysed in this study.

Samples	Regions	District	Virus Identified				
			SPLCV	SPLCD	PepYVMV	CLCuGeA	CLCuGeB
BFA310	Centre-Sud	Tiébélé	+	+	+	-	-
BFA313	Centre-Sud	Tiébélé	+	+	+	-	-
BFA351	Centre-Est	Bagré	+	+	-	+	-
BFA362	Centre-Est	Bagré	+	+	-	-	+
BFA942	Haut-Bassins	Samorogouan	+	+	+	-	+
BFA1094	Cascades	Banfora	+	+			+
BFA407	Boucle du Mouhoun	Di	+	+		+	
BFA662	Haut-Bassins	Kangala	+	+		+	

**Table 3:** Summary of identified viruses: accession numbers, genome lengths, coverage, sequencing depth, and phylogenetic links.

Isolates of This Study									Closest Relatives Retrieved from GenBank Organisms			
Isolates	District	Virus Identified	N° Accession	Length (bp)	% GC	Number of Reads	Coverage (%)	Mean Depth	Strains	Query Cover (%)	% of Identity	N° Accession
[BF:Bag:BFA351:16]	Bagré	CLCuGeA	PV405574	1353	38.9	1511	100	382.622	CLCuGeA Okra97B1	100	96.61	MK032296
[BF:Bag:BFA362:16]	Bagré	CLCuGeB	PV405578	1348	37.7	6	99.77	1.52819	CLCuGeB Okra1-1	100	97.78	FN554574
[BF:Di:BFA407:16]	Di	CLCuGeA	PV405575	1353	38.7	789	100	342.786	CLCuGeA Okra97B1	100	96.90	MK032296
[BF:Kan:BFA662:16]	Kangala	CLCuGeA	PV405576	1353	38.6	70	100	25.2	OkYCrCMA Njo5sp2	100	97.71	FN675287
[BF:Sam:BFA942:16]	Samorogouan	CLCuGeB	PV405579	1333	38.2	2	100	1.02326	CLCuGeB Okra3	100	96.52	FN554576
		PepYVMV	PV405580	2781	43.9	62039	100	5578.1	PepYVMV 796BE	100	99.17	MH778658
[BF:Ban:BFA1094:16]	Banfora	CLCuGeB	PV405577	1348	38.2	423	100	188.497	CLCuGeB Okra3	100	97.78	FN554576

## Data Availability

The datasets generated during and/or analyzed during the current study are available from the corresponding author on reasonable request. The complete genome sequences of the isolate obtained from Nanopore sequencing are available on GenBank under the accession numbers PV405574-PV405580.

## Supplementary Materials

The following supporting information can be downloaded at: https://www.mdpi.com/article/10.3390/v17091222/s1, Supplementary File S1: Supplementary Table S1: Pairwise nucleotide (nt) and amino acid (aa) sequence identity (%) between isolates characterised in this study by Nanopore and Sanger sequencing; Supplementary Table S2. Pairwise nucleotide (nt, lower triangle) and amino acid (aa, upper triangle) sequence identity (%) between PepYVMV isolate characterised in this study and homologous reference sequences; Supplementary Table S3. Pairwise nucleotide (nt, lower triangle) and amino acid (aa, upper triangle) sequence identity (%) between CLCuGeA isolates characterised in this study and homologous reference sequences; Supplementary Table S4. Pairwise nucleotide (nt, lower triangle) and amino acid (aa, upper triangle) sequence identity (%) between CLCuGeA isolates characterized in this study and homologous reference sequences.

## Abbreviations

The following abbreviations are used in this manuscript:

CLCuGeA	cotton leaf curl Gezira alphasatellite
CLCuGeB	cotton leaf curl Gezira betasatellite
CP	coat protein
HTS	high-throughput sequencing
MeCSV	melon chlorotic spot virus
MP	movement protein
OkLCMB	okra leaf curl Mali virus betasatellite
ONT	Oxford Nanopore Technologies
ORF	Open Reading Frame
OYCrCMA	okra yellow crinkle Cameroon alphastellite
PaLCGeB	papaya leaf curl Gezira betasatellite
PepYVMV	pepper yellow vein Mali virus
RCA	Rolling Circle Amplification
REn	Replication enhancer
Rep	Replication protein
SCR	satellite conserved region
SPCSV	sweet potato chlorotic stunt virus
SPFMV	sweet potato feathery mottle virus
SPLCD3	sweet potato leaf curl deltasatellite 3
SPLCA	sweet potato leaf curl alphasatellite
SPLCV	sweet potato leaf curl virus
ToCV	tomato chlorosis virus
ToLCV	tomato leaf curl virus
ToLCMV	tomato leaf curl Mali virus
ToYLCMV	tomato yellow leaf curl Mali virus
TrAp	Transcriptional activator protein
