# Supplementary material for: Insights into Novel Viral Threats in Sweetpotato from Burkina Faso: Characterisation of Unexplored Pathogens
Source: Viruses. 2025 Sep 7;17(9):1222. doi: 10.3390/v17091222 (PMC12474108; doi:10.3390/v17091222)
Supplement: Supplementary file 1 [file viruses-17-01222-s001.zip › viruses-3846837-supplementary.pdf]

Supplementary Table S1. Pairwise nucleotide (nt) and amino acid (aa) sequence identity (%) between isolates characterized in this study by Nanopore and Sanger sequencing

Isolate comparison	ORF compared	nt identity (%)	aa identity (%)
BFA942_Sanger vs BFA942_Nanopore	Partiel Coat protein	99.7	99.65

Supplementary Table S2. Pairwise nucleotide (nt, lower triangle) and amino acid (aa, upper triangle) sequence identity (%) between PepYVMV isolate characterized in this study and homologous reference sequences.

	MH778658	PV405580	FM876851	MH778662	FN555171	MH778651	MH460532	OR483374	KY271077	MH778685	ON367442	ON367443	MH778673	NC_005347	AM691547	AM691549	FN555173	MN372224	EU635776	LM651400	AY502936	Outgroup
MH778658		99.28	98.60	98.31	98.99	97.91	97.77	96.47	96.11	96.08	96.15	95.72	95.79	95.97	92.30	92.22	95.64	91.22	79.85	79.81	78.35	59.32
PV405580	99.32		98.31	98.16	98.81	97.70	97.55	96.33	95.82	95.79	95.93	95.43	95.50	95.68	92.04	91.90	95.43	90.96	79.74	79.73	78.03	59.16
FM876851	98.60	98.34		98.70	99.14	97.98	97.91	96.29	96.51	96.47	96.76	96.19	96.19	96.22	92.52	92.37	96.19	91.40	79.96	79.81	78.29	59.36
MH778662	98.31	98.20	98.70		98.85	97.91	97.80	96.40	95.93	95.90	96.19	95.61	95.61	95.75	92.12	91.90	95.65	91.18	79.85	79.56	78.22	59.24
FN555171	98.99	98.85	99.14	98.85		98.31	98.16	96.73	96.33	96.29	96.51	96.01	96.01	96.19	92.52	92.34	96.01	91.58	79.99	79.92	78.22	59.28
MH778651	97.91	97.73	97.98	97.91	98.31		99.06	96.22	95.93	95.90	96.65	95.65	95.57	95.68	92.12	91.90	95.57	91.00	80.17	79.92	78.36	59.12
MH460532	97.77	97.59	97.91	97.80	98.16	99.06		95.97	95.79	95.75	96.83	95.54	95.39	95.50	92.01	91.76	95.39	90.97	80.14	79.81	78.47	59.20
OR483374	96.47	96.36	96.29	96.40	96.73	96.22	95.97		95.32	95.29	95.25	95.00	95.07	95.18	91.69	91.47	95.03	90.72	80.21	79.46	77.93	59.04
KY271077	96.11	95.86	96.51	95.93	96.33	95.93	95.79	95.32		99.96	98.56	99.32	98.92	98.03	94.33	94.22	98.13	92.79	80.40	79.94	78.68	59.12
MH778685	96.08	95.82	96.47	95.90	96.29	95.90	95.75	95.29	99.96		98.52	99.28	98.89	97.99	94.29	94.19	98.10	92.75	80.37	79.90	78.65	59.12
ON367442	96.15	95.97	96.76	96.19	96.51	96.65	96.83	95.25	98.56	98.52		98.16	97.55	96.98	93.27	93.16	97.12	91.72	80.17	79.67	78.43	59.08
ON367443	95.72	95.46	96.19	95.61	96.01	95.65	95.54	95.00	99.32	99.28	98.16		98.46	97.56	93.90	93.79	97.56	92.28	80.44	79.83	78.68	59.16
MH778673	95.79	95.53	96.19	95.61	96.01	95.57	95.39	95.07	98.92	98.89	97.55	98.46		97.74	93.97	93.90	97.74	92.57	80.30	79.90	78.65	59.20
NC_005347	95.97	95.71	96.22	95.75	96.19	95.68	95.50	95.18	98.03	97.99	96.98	97.56	97.74		96.05	96.02	98.42	93.61	80.19	80.01	78.43	59.12
AM691547	92.30	92.08	92.52	92.12	92.52	92.12	92.01	91.69	94.33	94.29	93.27	93.90	93.97	96.05		96.05	94.47	89.66	78.81	78.35	77.49	59.20
AM691549	92.22	91.93	92.37	91.90	92.34	91.90	91.76	91.47	94.22	94.19	93.16	93.79	93.90	96.02	96.05		94.44	89.63	78.81	78.53	77.24	59.04
FN555173	95.64	95.46	96.19	95.65	96.01	95.57	95.39	95.03	98.13	98.10	97.12	97.56	97.74	98.42	94.47	94.44		94.04	80.12	79.86	78.50	59.00
MN372224	91.22	91.00	91.40	91.18	91.58	91.00	90.97	90.72	92.79	92.75	91.72	92.28	92.57	93.61	89.66	89.63	94.04		78.67	78.43	77.17	58.76
EU635776	79.85	79.77	79.96	79.85	79.99	80.17	80.14	80.21	80.40	80.37	80.17	80.44	80.30	80.19	78.81	78.81	80.12	78.67		89.39	77.79	57.90
LM651400	79.81	79.76	79.81	79.56	79.92	79.92	79.81	79.46	79.94	79.90	79.67	79.83	79.90	80.01	78.35	78.53	79.86	78.43	89.39		77.67	57.45
AY502936	78.35	78.05	78.29	78.22	78.22	78.36	78.47	77.93	78.68	78.65	78.43	78.68	78.65	78.43	77.49	77.24	78.50	77.17	77.79	77.67		58.31
Outgroup	59.39	59.26	59.43	59.31	59.35	59.19	59.27	59.11	59.19	59.19	59.15	59.23	59.27	59.19	59.27	59.11	59.07	58.83	57.97	57.51	58.38	

Supplementary Table S3. Pairwise nucleotide (nt, lower triangle) and amino acid (aa, upper triangle) sequence identity (%) between CLCuGeA isolates characterized in this study and homologous reference sequences.

	PV405574	PV405575	MK032296	PV405576	FN675287	FN675284	FN554583	MK032295	KC763631	ON756231	KC763632	MN027204	MT316193	HE858192	MW779543	JX050199
PV405574		98.3	96.6	95.71	95.19	94.97	95.49	95.56	92.23	91.93	92.3	94.53	94.01	49.77	51.22	36.57
PV405575	98.3		96.9	95.93	95.34	95.19	95.71	95.78	92.6	92.3	92.45	94.83	94.31	49.92	51.37	36.57
MK032296	96.6	96.9		97.12	96.3	96.01	97.04	97.04	92.6	92.3	92.6	95.49	94.98	50.38	51.45	36.9
PV405576	95.71	95.93	97.12		97.71	96.97	96.89	97.41	93.04	92.74	92.96	96.23	95.64	50.61	51.83	36.19
FN675287	95.19	95.34	96.3	97.71		96.23	96.23	97.12	92.52	92.07	92.37	95.57	95.12	50.31	51.76	36.57
FN675284	94.97	95.19	96.01	96.97	96.23		96.6	96.15	92.52	92.07	92.37	95.2	94.6	50.31	51.07	36.13
FN554583	95.49	95.71	97.04	96.89	96.23	96.6		97.93	92.81	92.67	92.74	95.71	95.2	50.99	51.83	36.88
MK032295	95.56	95.78	97.04	97.41	97.12	96.15	97.93		92.74	92.59	92.81	95.93	95.34	50.53	51.91	36.65
KC763631	92.23	92.6	92.6	93.04	92.52	92.52	92.81	92.74		99.41	98.22	93.78	93.26	50.08	52.06	36.75
ON756231	91.93	92.3	92.3	92.74	92.07	92.07	92.67	92.59	99.41		97.88	93.34	92.82	49.59	50.85	36.71
KC763632	92.3	92.45	92.6	92.96	92.37	92.37	92.74	92.81	98.22	97.88		93.63	93.12	49.63	51.26	36.79
MN027204	94.53	94.83	95.49	96.23	95.57	95.2	95.71	95.93	93.78	93.34	93.63		99.11	51.26	52.1	37
MT316193	94.01	94.31	94.98	95.64	95.12	94.6	95.2	95.34	93.26	92.82	93.12	99.11		51.33	52.33	37.08
HE858192	49.77	49.92	50.38	50.61	50.31	50.31	50.99	50.53	50.08	49.59	49.63	51.26	51.33		70.8	36.21
MW779543	51.22	51.37	51.45	51.83	51.76	51.07	51.83	51.91	52.06	50.85	51.26	52.1	52.33	70.8		35.12
JX050199	36.57	36.57	36.9	36.19	36.57	36.13	36.88	36.65	36.75	36.71	36.79	37	37.08	36.21	35.12	

Supplementary Table S4. Pairwise nucleotide (nt, lower triangle) and amino acid (aa, upper triangle) sequence identity (%) between CLCuGeA isolates characterized in this study and homologous reference sequences.

	PV405577	PV405579	FM164728	FN554576	FN554574	PV405578	FJ469628	MK032305	FN554573	MK032303	MK456609	MT316190	PP093771	ON756227	MN508949	MZ911859	AY044142	MW779543
PV405577		99.62	97.64	97.85	97.77	97.77	96.88	96.58	97.03	96.35	88.38	88.85	85.99	87.67	85.63	83.82	92.72	43.13
PV405579	99.62		97.48	97.6	97.52	97.37	96.47	96.17	96.62	95.93	88.08	88.48	85.59	87.28	85.46	83.63	92.26	43.45
FM164728	97.64	97.48		98.71	98.02	97.26	96.28	95.81	96.2	95.65	88.64	88.73	85.25	87.12	85.04	83.19	92.54	43.45
FN554576	97.85	97.6	98.71		98.14	97.4	96.36	95.99	96.36	95.83	88.77	89.01	85.92	87.68	85.64	83.83	92.79	43.01
FN554574	97.77	97.52	98.02	98.14		98.21	97.03	96.8	97.1	96.64	89.14	89.76	86.44	87.97	85.92	83.98	93.68	43.16
PV405578	97.77	97.37	97.26	97.4	98.21		97.33	96.87	97.25	96.72	88.9	89.37	86.12	87.34	85.22	83.41	93.3	42.97
FJ469628	96.88	96.47	96.28	96.36	97.03	97.33		98.07	98.44	97.32	89.15	89.62	85.92	87.68	85.25	83.74	93.17	43.01
MK032305	96.58	96.17	95.81	95.99	96.8	96.87	98.07		98.51	97.62	88.28	89.06	85.51	87.02	84.76	83.4	92.71	42.41
FN554573	97.03	96.62	96.2	96.36	97.1	97.25	98.44	98.51		98.51	88.68	89.46	86.3	87.67	85.32	83.96	93.09	42.58
MK032303	96.35	95.93	95.65	95.83	96.64	96.72	97.32	97.62	98.51		88.34	89.12	86.18	87.39	85.04	83.68	92.92	43.03
MK456609	88.38	88.08	88.64	88.77	89.14	88.9	89.15	88.28	88.68	88.34		96	88.43	88.67	85.05	85.17	89.54	42.47
MT316190	88.85	88.48	88.73	89.01	89.76	89.37	89.62	89.06	89.46	89.12	96		88.82	89.07	85.53	85.33	90.38	42.92
PP093771	85.99	85.59	85.25	85.92	86.44	86.12	85.92	85.51	86.3	86.18	88.43	88.82		96.23	92.02	92.52	87.94	41.94
ON756227	87.67	87.28	87.12	87.68	87.97	87.34	87.68	87.02	87.67	87.39	88.67	89.07	96.23		95.56	94.11	89.18	41.63
MN508949	85.69	85.53	85.11	85.7	85.99	85.29	85.32	84.82	85.38	85.11	85.12	85.59	92.09	95.56		95.49	87.04	41.15
MZ911859	83.82	83.63	83.19	83.83	83.98	83.41	83.74	83.4	83.96	83.68	85.17	85.33	92.52	94.11	95.56		85.08	41.58
AY044142	92.72	92.26	92.54	92.79	93.68	93.3	93.17	92.71	93.09	92.92	89.54	90.38	87.94	89.18	87.11	85.08		43.49
MW779543	43.13	43.45	43.45	43.01	43.16	42.97	43.01	42.41	42.58	43.03	42.47	42.92	41.94	41.63	41.18	41.58	43.49	
